# Esomeprazole Decreases Soluble Fms-like Tyrosine Kinase-1 in Preeclamptic Pregnancy in Rats

**DOI:** 10.3390/ijms27073105

**Published:** 2026-03-29

**Authors:** Maria Luiza Santos da Silva, Cristal de Jesus Toghi, Augusto Antunes Fraga da Silva, Hellen Cristiny Cavalcanti de Souza, Beatriz Dragoneti Jorge, Helio Kushima, Flávia Bessi Constantino Colenci, Guilherme Henrique Marchi Salvador, Marcos Roberto de Mattos Fontes, Carlos Alexandre Henrique Fernandes, Carlos Alan Dias-Junior

**Affiliations:** Department of Biophysics and Pharmacology, Institute of Biosciences, Sao Paulo State University (UNESP), Botucatu 18618-689, SP, Brazil; mls.silva@unesp.br (M.L.S.d.S.); c.toghi@unesp.br (C.d.J.T.); antunes.fraga@unesp.br (A.A.F.d.S.); hellen.cristiny@unesp.br (H.C.C.d.S.); beatriz.dragoneti@unesp.br (B.D.J.); helio.kushima@unesp.br (H.K.); flavia.bessi@unesp.br (F.B.C.C.); guilherme.salvador@unesp.br (G.H.M.S.); marcos.fontes@unesp.br (M.R.d.M.F.); carlos.fernandes@unesp.br (C.A.H.F.)

**Keywords:** preeclampsia, esomeprazole, nitric oxide, placental ischemia, endothelial dysfunction

## Abstract

Preeclampsia is a hypertensive disorder of pregnancy associated with elevated levels of soluble fms-like tyrosine kinase-1 (sFlt-1) and reduced nitric oxide (NO) bioavailability. Esomeprazole (ESO), a proton pump inhibitor (PPI) considered safe during pregnancy, has been proposed to reduce sFlt-1 levels in vitro. This study evaluated the effects of ESO in pregnant rats subjected to reduced uterine perfusion pressure (RUPP), a well-established model of preeclampsia. Pregnant rats received saline (Preg) or ESO (Preg+ESO), while RUPP-operated rats received saline (RUPP) or ESO (RUPP+ESO). At gestational day 21, maternal blood pressure was elevated in the Preg+ESO, RUPP, and RUPP+ESO groups compared with Preg, and ESO did not attenuate RUPP-induced hypertension. Fetal and placental weights were reduced in the RUPP group, whereas ESO increased placental weight in Preg+ESO and RUPP+ESO groups. Gastric pH was elevated by ESO, confirming reduced gastric acidity. Plasma sFlt-1 levels were increased in RUPP and significantly reduced by ESO in RUPP+ESO rats. NO metabolites (NOx) were decreased in RUPP but were unaffected by treatment. Endothelium-dependent relaxation was impaired in the RUPP and RUPP+ESO groups. In conclusion, ESO did not prevent hypertension or endothelial dysfunction, but reduced circulating sFlt-1, suggesting a partial modulatory effect on angiogenic imbalance in experimental preeclampsia.

## 1. Introduction

Preeclampsia affects 5–8% of pregnancies and is a major contributor to maternal mortality worldwide [1]. Preeclampsia is a disorder characterized by maternal hypertension from the 20th week of gestation, often accompanied by proteinuria, fetal and placental growth restrictions, and systemic vascular endothelial inflammation-activation-dysfunction [2]. Importantly, there are no effective medications or preventive strategies for preeclampsia [3].

The pathogenesis of preeclampsia is still not fully understood. Previous studies have proposed that, as a consequence of impaired uterine artery remodeling in early pregnancy, leading to reduced placental perfusion, the preeclamptic placenta exhibits oxidative stress and the release of anti-angiogenic factors [2], including sFlt-1 [1].

sFlt-1 is a non-membrane, alternatively spliced form of vascular endothelial growth factor (VEGF) receptor-1 (VEGFR-1) that binds to VEGF and placental growth factor (PlGF), reducing their bioavailability and antagonizing pro-angiogenic signaling, thereby impairing vascular homeostasis in conditions such as preeclampsia [4,5,6]. Elevated circulating sFlt-1 levels have also been implicated in endothelial dysfunction by disrupting VEGF-mediated NO synthesis and endothelial NO synthase (eNOS) phosphorylation pathways, which may contribute to increases in blood pressure [7].

During pregnancy, PPIs are frequently prescribed and are generally considered safe, including during the first trimester [8,9,10,11,12]. ESO, a widely used PPI, is indicated for the treatment of gastrointestinal disorders such as gastroesophageal reflux and peptic ulcer disease [10,11]. Despite their favorable safety profile, emerging evidence suggests that the use of PPIs may reduce circulating levels of NO metabolites [13]. This is of particular concern because gastric acidity is required for the conversion of dietary nitrate to nitrite, a key step in the alternative endothelium-independent pathway of NO generation [13]. Since this pathway contributes to the maintenance of systemic NO bioavailability [13], the use of PPIs could potentially impair NO formation during pregnancy [14,15].

Conversely, recent studies have highlighted a potential therapeutic role for PPIs in preeclampsia. Experimental data and clinical trials indicate that PPIs reduce circulating levels of sFlt-1 and may be used for the prevention of preeclampsia [16]. However, whether ESO reduces sFlt-1 in placental ischemia-induced preeclampsia has not yet been investigated.

To address this question, we used the RUPP model in pregnant rats, a well-established experimental model of preeclampsia [17]. RUPP reproduces key features of the disorder, including increased maternal blood pressure, endothelial dysfunction, and reduced fetal and placental weights [17]. Therefore, the present study aimed to evaluate the physiological effects of ESO during healthy pregnancy and to investigate its impact in experimental preeclampsia.

## 2. Results

### 2.1. Effects of Esomeprazole Treatment on Blood Pressure Parameters at Gestational Day 21

**Figure 1:**
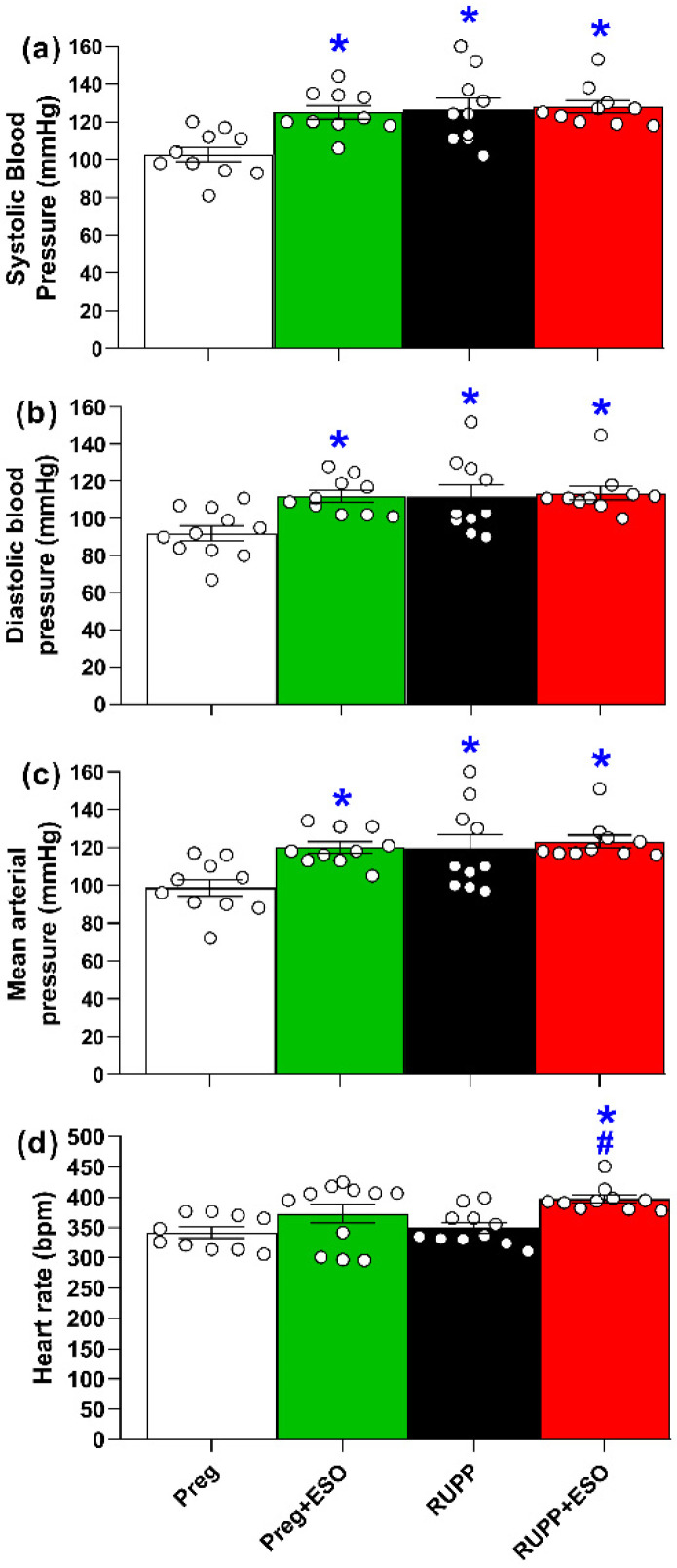
Effects of esomeprazole treatment on maternal systolic blood pressure (**a**), diastolic blood pressure (**b**), mean arterial pressure (**c**), and heart rate (**d**) measured on gestational day 21 in the Preg, Preg+ESO, RUPP, and RUPP+ESO groups (*n* = 8–10 animals per group). Data are presented as mean ± SEM. * *p* < 0.05 vs. Preg group; ^#^ *p* < 0.05 vs. RUPP group.

At gestational day 21, maternal systolic, diastolic, and mean arterial pressures were significantly elevated in the Preg+ESO, RUPP, and RUPP+ESO rats compared with the Preg group (Figure 1a–c; Table 1). ESO treatment did not attenuate the RUPP-induced elevations in arterial blood pressure (Figure 1; Table 1).

Additionally, heart rate was significantly increased in the RUPP+ESO group compared with the Preg and RUPP groups (Figure 1d; Table 1).

### 2.2. Effects of Esomeprazole Treatment on Fetal Parameters

**Figure 2:**
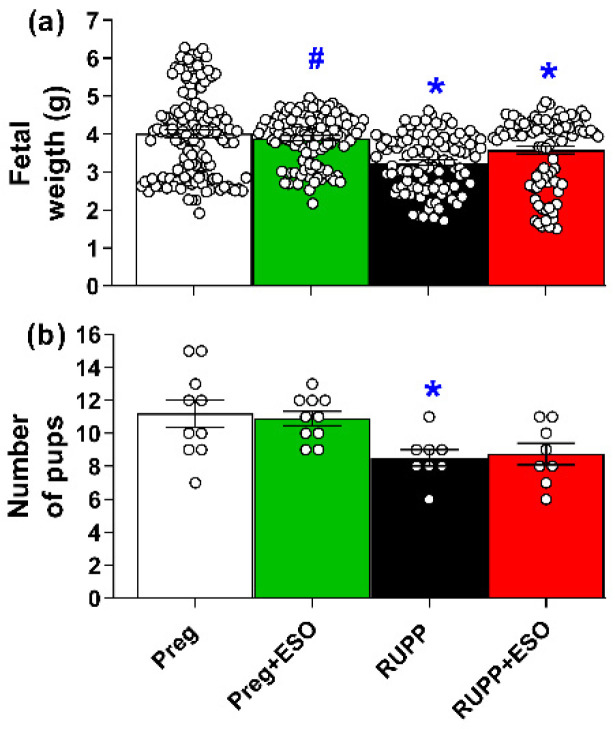
Effects of esomeprazole treatment on fetal weight (**a**) and number of pups (**b**) in the Preg, Preg+ESO, RUPP, and RUPP+ESO groups (*n* = 8–10 animals per group). Data are presented as mean ± SEM. * *p* < 0.05 vs. Preg group; ^#^ *p* < 0.05 vs. RUPP group.

Fetal weight was reduced in the RUPP and RUPP+ESO compared with the Preg group (Figure 2a; Table 1). Fetal weight in the Preg+ESO group did not differ from Preg, but it was significantly higher compared with the RUPP group.

The number of pups per litter was significantly decreased in the RUPP group compared with the Preg rats. No significant differences were observed between Preg+ESO and RUPP+ESO groups (Figure 2b; Table 1).

### 2.3. Effect of Esomeprazole Treatment on Placental Weight

**Figure 3:**
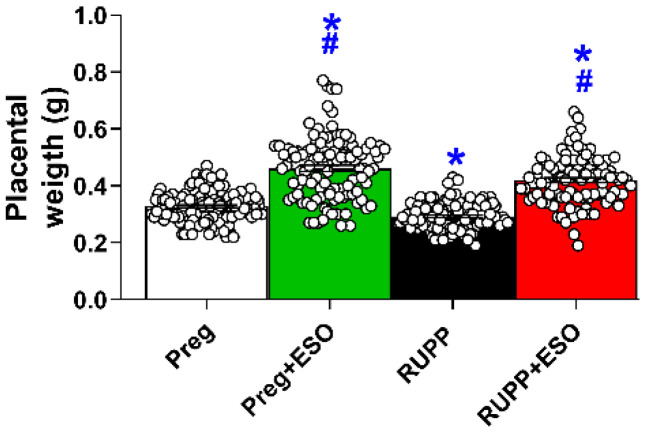
Effects of esomeprazole treatment on placental weight in the Preg, Preg+ESO, RUPP, and RUPP+ESO groups (*n* = 8–10 animals per group). Data are presented as mean ± SEM. * *p* < 0.05 vs. Preg group; ^#^ *p* < 0.05 vs. RUPP group.

Placental weight was also significantly reduced in the RUPP compared with the Preg group (Figure 3; Table 1). ESO treatment increased placental weight in both Preg+ESO and RUPP+ESO groups compared with the Preg and RUPP groups.

### 2.4. Effect of Esomeprazole Treatment on Placental Efficiency

**Figure 4:**
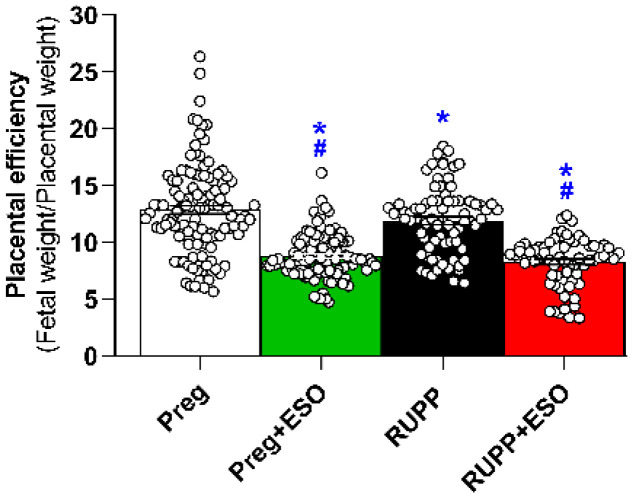
Effects of esomeprazole treatment on placental efficiency in the Preg, Preg+ESO, RUPP, and RUPP+ESO groups (*n* = 8–10 animals per group). Placental efficiency was calculated as the ratio between fetal weight and placental weight. Data are presented as mean ± SEM. * *p* < 0.05 vs. Preg group; ^#^ *p* < 0.05 vs. RUPP group.

Placental efficiency was significantly reduced in the RUPP group compared with the Preg group (Figure 4; Table 1). ESO treatment significantly decreased placental efficiency in both Preg+ESO and RUPP+ESO groups compared with the Preg and RUPP groups, respectively (Figure 4; Table 1).

### 2.5. Effect of Esomeprazole Treatment on Gastric pH

**Figure 5:**
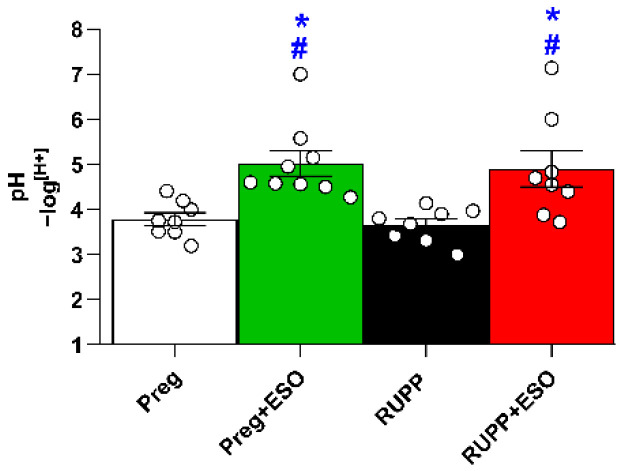
Effects of esomeprazole treatment on gastric pH in the Preg, Preg+ESO, RUPP, and RUPP+ESO groups (*n* = 8–10 animals per group). Data are presented as mean ± SEM. * *p* < 0.05 vs. Preg group; ^#^ *p* < 0.05 vs. RUPP group.

Gastric pH was significantly elevated in the Preg+ESO and RUPP+ESO compared with the Preg and RUPP groups, indicating effective gastric acid suppression by ESO in pregnant rats (Figure 5; Table 1).

### 2.6. Effects of Esomeprazole Treatment on Circulating sFlt-1

**Figure 6:**
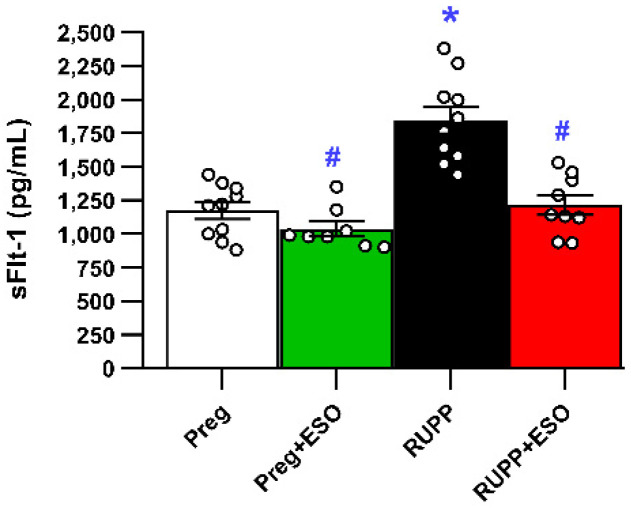
Effects of esomeprazole treatment on circulating sFlt-1 levels in plasma from Preg, Preg+ESO, RUPP, and RUPP+ESO groups (*n* = 8–10 animals per group). Data are presented as mean ± SEM. * *p* < 0.05 vs. Preg group; ^#^ *p* < 0.05 vs. RUPP group.

Plasma sFlt-1 levels were increased in the RUPP group compared with the Preg, Preg+ESO, and RUPP+ESO groups (Figure 6; Table 1). ESO treatment significantly reduced circulating sFlt-1 levels in plasma from RUPP+ESO rats compared with the untreated RUPP animals (Figure 6; Table 1).

### 2.7. Assessment of Circulating VEGF Levels

**Figure 7:**
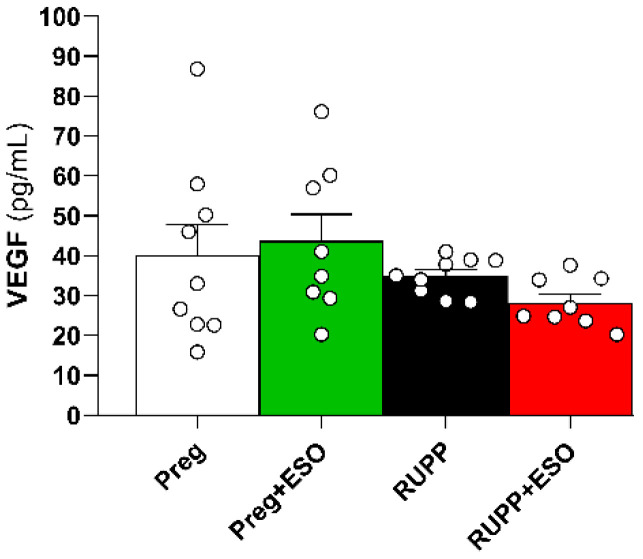
Assessment of VEGF levels in plasma from Preg, Preg+ESO, RUPP, and RUPP+ESO groups (*n* = 8–10 animals per group). Data are presented as mean ± SEM. No statistically significant differences were observed among the four groups.

Plasma VEGF levels did not differ significantly among the four groups (Figure 7; Table 1).

### 2.8. Assessment of sFlt-1/VEGF Ratio

**Figure 8:**
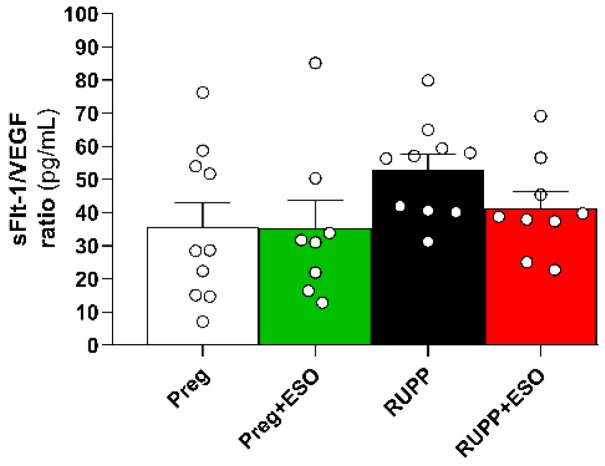
Assessment of sFlt-1/VEGF ratio in plasma from Preg, Preg+ESO, RUPP, and RUPP+ESO groups (*n* = 8–10 animals per group). The ratio reflects the balance between sFlt-1 and VEGF. Data are presented as mean ± SEM.

The circulating sFlt-1/VEGF ratio, which reflects the balance between anti-angiogenic sFlt-1 and pro-angiogenic VEGF, was calculated for all experimental groups. No statistically significant differences were detected among the Preg, Preg+ESO, RUPP, and RUPP+ESO groups (Figure 8; Table 1).

### 2.9. Assessment of Circulating ADMA Levels

**Figure 9:**
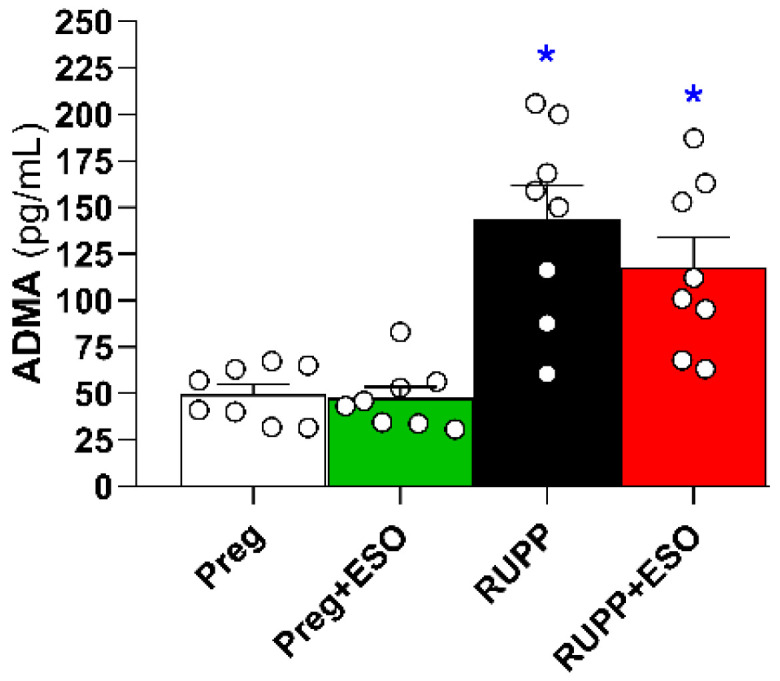
Assessment of circulating ADMA levels in plasma from Preg, Preg+ESO, RUPP, and RUPP+ESO groups (*n* = 8–10 animals per group). Data are presented as mean ± SEM. * *p* < 0.05 vs. Preg group.

Plasma asymmetric dimethylarginine (ADMA) levels were significantly increased in the RUPP and RUPP+ESO groups compared with the Preg and Preg+ESO groups (Figure 9; Table 1). ESO treatment did not modify ADMA levels in RUPP+ESO rats.

### 2.10. Effect of Esomeprazole Treatment on Plasma NO Metabolites (NOx)

**Figure 10:**
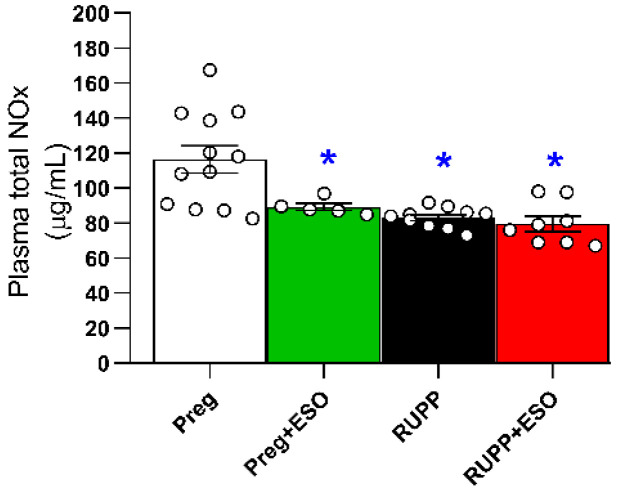
Effects of esomeprazole treatment on plasma NO metabolites (NOx) in the Preg, Preg+ESO, RUPP, and RUPP+ESO groups (*n* = 8–10 animals per group). Data are presented as mean ± SEM. * *p* < 0.05 vs. Preg group.

Plasma NO metabolites (nitrate and nitrite) levels were significantly reduced in the Preg+ESO, RUPP, and RUPP+ESO groups compared with the Preg rats. No significant differences among Preg+ESO. RUPP and RUPP+ESO groups were observed (Figure 10; Table 1). ESO treatment significantly reduced plasma NO metabolites levels in Preg+ESO rats compared with the Preg group (Figure 10; Table 1).

### 2.11. Effects of Esomeprazole Treatment on Acetylcholine-Induced Relaxation

**Figure 11:**
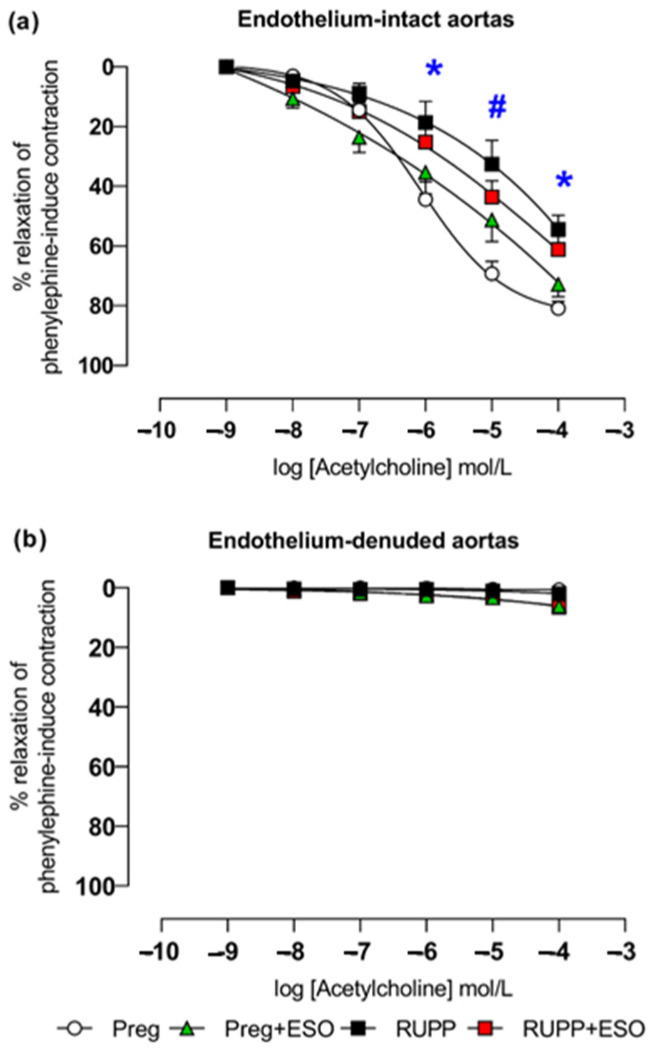
Effects of esomeprazole treatment on acetylcholine-induced relaxation in endothelium-intact (**a**) and endothelium-denuded (**b**) thoracic aortic rings from the Preg, Preg+ESO, RUPP, and RUPP+ESO groups (*n* = 8–10 animals per group). Data are presented as mean ± SEM. * *p* < 0.05 for Preg and Preg+ESO vs. RUPP and RUPP+ESO groups; ^#^ *p* < 0.05 for Preg vs. Preg+ESO, RUPP, and RUPP+ESO groups.

In thoracic aortic rings with intact endothelium pre-contracted with phenylephrine, acetylcholine-induced relaxation was significantly impaired in the RUPP and RUPP+ESO groups compared with the Preg controls at concentrations of 10^−6^, 10^−5^, and 10^−4^ M (Figure 11a; Table 1).

Aortic rings from Preg+ESO rats showed reduced relaxation only at 10^−5^ M compared with Preg control, and at 10^−6^ and 10^−4^ concentrations in comparison with the RUPP and RUPP+ESO groups.

In endothelium-denuded rings, acetylcholine-induced responses did not differ among any of the groups (Figure 11b).

### 2.12. Molecular Docking and Molecular Dynamics (MD) Simulations Between sFlt-1 and Esomeprazole

Among the top docking poses for the D2 and D2–D3 constructs, ESO interacted at the interface between immunoglobulin-like domains and VEGF, with predicted binding energies of approximately −5.0 kcal/mol. However, in all cases, the ligand failed to remain at the predicted sites during MD simulations; in the majority of the trajectories, ESO dissociated from the protein within the first 50 ns of simulations (Supplementary Figure S1A–D).

For the Flt-1 D1–D6 model, a single docking pose was identified at domain D5, located at the dimer interface between sFlt-1 chains (Supplementary Figure S1E), with a somewhat more favorable predicted affinity (~−10 kcal/mol); nonetheless, the ligand also rapidly dissociated during MD simulations.

## 3. Discussion

In the present study, the RUPP model reproduced key features commonly associated with preeclampsia, including elevated maternal blood pressure, reduced fetal and placental weights, increased circulating sFlt-1 levels, decreased NO metabolites, and impaired endothelium-dependent vasodilation, as demonstrated by attenuated acetylcholine-induced relaxation [17]. These findings are consistent with previous characterizations of the model and support the presence of placental ischemia–associated angiogenic imbalance and endothelial dysfunction [17].

Although ESO treatment reduced circulating sFlt-1 levels in RUPP+ESO animals, it did not attenuate hypertension or fully restore endothelial function. In addition, increased blood pressure was observed in pregnant rats treated with ESO (Preg+ESO group). While this finding should be interpreted with caution, it raises the possibility that ESO may influence vascular regulation under certain conditions, consistent with clinical and experimental studies linking chronic PPI use to an increased risk of hypertension [18]. An epidemiological large cohort study conducted by Soliman et al. (2025) reported that PPI use was associated with 17% higher risk of hypertension for menopausal women compared with nonuse [18]. In addition, ESO did not attenuate blood pressure in a mouse model of L-NAME-induced hypertension [9].

One potential mechanism underlying the hemodynamic findings involves NO bioavailability. In addition to the classical L-arginine–eNOS pathway, NO can be generated through the nitrate–nitrite–NO pathway, in which dietary nitrate is reduced to nitrite by oral bacteria and subsequently converted to NO in the acidic gastric environment [19,20]. Because PPIs increase gastric pH, suppression of gastric acidity may reduce nitrite conversion to NO, potentially decreasing systemic NO levels [21]. Clinical data have shown that pretreatment with ESO can attenuate the antihypertensive effect of oral nitrite in humans [22], supporting the biological plausibility of this mechanism. Consistently, circulating NO metabolites were reduced in the ESO-treated groups in the present study. The modulation of gastric pH by ESO treatment is already well documented in the literature, given its mechanism of action used to reduce gastric discomfort caused by increased stomach acidity [23]. Accordingly, the animals that received ESO exhibited reduced acidity of gastric contents [24]. Since the conversion of nitrite to bioactive NO in the stomach depends on an acidic environment, suppression of gastric acid production may have limited this alternative pathway of NO generation in the present study. While long-term PPI exposure has been associated with changes in endothelial function, the impact of suppression of the nitrate–nitrite–NO pathway may occur more rapidly following gastric acid inhibition [18]. Thus, impaired NO bioavailability could contribute to vascular dysfunction and explain the absence of blood pressure-lowering effects in RUPP+ESO rats.

Moreover, our findings demonstrate relevant clinical implications considering the widespread use of PPIs during pregnancy. In the present study, ESO increased blood pressure, reduced NO metabolites in plasma, and impaired acetylcholine-induced relaxation (10^−5^ M) in aortas of Preg+ESO rats. Since NO is essential for physiological vascular adaptations during pregnancy [25], these results suggest that ESO use in healthy pregnancies may interfere with critical regulatory pathways of maternal vascular function. Although extrapolation to humans requires caution, the use of a translationally relevant model reinforces the hypothesis that ESO use could influence physiological balance, particularly in pregnant women predisposed to hypertensive disorders [9,18].

The increase in heart rate observed in the RUPP+ESO group may represent a compensatory mechanism in response to hemodynamic changes. Since ESO raises gastric pH, it may have interfered with the alternative NO formation pathway, further reducing its bioavailability [13]. The reduced plasma NO metabolites in the RUPP+ESO group may explain, at least in part, the sympathetic activation, leading to tachycardia [26]. Although we did not examine the changes in peripheral vascular resistance or markers of sympathetic activation, there is previous evidence that NO acts as a crucial neuromodulator in the cardiovascular system, regulating blood pressure and flow by restraining sympathetic outflow and facilitating parasympathetic activity within the central and peripheral nervous system [26]. Therefore, the elevated heart rate in ESO-treated RUPP animals could reflect an adaptation to maintain cardiac output in the face of the increased vascular resistance, a characteristic sign of the RUPP model of preeclampsia.

Acetylcholine-induced relaxation was significantly impaired in the RUPP group and was not fully restored by ESO treatment. This functional result is consistent with the observed reduction in circulating NO metabolites in ESO-treated animals and further supports the possibility of compromised NO bioavailability. Endothelium-dependent relaxation in response to acetylcholine is largely mediated by endothelial NO production. Aligned with previous reports indicating that PPIs may influence NO-related pathways [18], Ghebremariam et al. (2013) reported that PPIs—including ESO—can increase plasma levels of ADMA, an endogenous inhibitor of NOS synthase, thereby reducing NO production and impairing vascular relaxation [21]. Similarly, Arafah and collaborators (2018) demonstrated that pantoprazole decreased acetylcholine-induced aortic relaxation in mice, indicating an adverse effect on endothelium-dependent vasodilation [27].

ADMA is an endogenous competitive inhibitor of eNOS that reduces NO production by limiting L-arginine availability at the catalytic site of the enzyme [28]. Elevated ADMA has been associated with endothelial dysfunction, increased vascular resistance, and adverse cardiovascular outcomes [29]. In the context of preeclampsia, increased ADMA concentrations have been consistently reported by clinical evidence demonstrating increased circulating ADMA in preeclamptic women compared with normotensive pregnancies [30]. Importantly, ADMA levels are regulated primarily by dimethylarginine dimethylaminohydrolase (DDAH), the enzyme responsible for its degradation [21]. Experimental studies suggest that PPIs may interfere with ADMA metabolism by inhibiting DDAH activity, thereby promoting ADMA accumulation and further limiting NO production [21,31]. This mechanism can provide a link between PPIs exposure and endothelial dysfunction. In the present study, the elevated ADMA levels in RUPP+ESO animals, together with reduced circulating NO metabolites and incomplete recovery of acetylcholine-induced relaxation, suggest that ESO did not restore the methylarginine–eNOS pathway, which may contribute to its limited vascular benefits in this model.

Another important aspect to consider is that the vascular effects of ESO may depend on the oxidative and inflammatory environment present in preeclampsia. Previous studies have shown that PPIs can exert antioxidant effects by reducing reactive oxygen species (ROS) formation and improving endothelial viability under stress conditions [9]. Ghebremariam (2013) also reported that ESO enhances eNOS phosphorylation and limits oxidative injury in cultured endothelial cells exposed to inflammatory cytokines [21]. However, in the RUPP model, where oxidative stress and inflammation are already markedly elevated [32], these potential protective effects might be insufficient to counterbalance the pronounced endothelial dysfunction. As PPIs can increase ADMA concentrations [21], this mechanism could further impair NO bioavailability and contribute to the incomplete recovery of vascular relaxation observed in the ESO-treated RUPP group. Interestingly, other studies have found that ESOcan improve vascular relaxation in specific conditions, such as in obese pregnant mice treated with L-NAME [9]. In the RUPP model, the lack of complete recovery of acetylcholine-induced relaxation may reflect complex interactions between ESO and the NO pathways, and further studies are needed to clarify the underlying mechanisms.

Experimental and clinical studies have consistently demonstrated that elevated circulating sFlt-1 levels correlate with reduced NO bioavailability, impaired endothelium-dependent vasodilation, and hypertension severity in preeclamptic pregnancies [5,6,7]. Indeed, we observed that ESO treatment reduced circulating sFlt-1 levels in RUPP rats, consistent with previous studies showing that PPI use decreased sFlt-1 in pregnant women [33]. However, this biochemical improvement was not accompanied by hemodynamic recovery or restoration of endothelial function. Specifically, reductions in sFlt-1 did not translate into consistent improvements in blood pressure or acetylcholine-induced vasodilation. The present dataset may be explained, at least in part, by the reduced NO metabolites and increased levels of ADMA, as previously reported [34]. This apparent dissociation between sFlt-1 reduction and functional recovery may be explained, at least in part, by the concurrent reduction in NO metabolites. Taken together, our findings suggest that restoring NO bioavailability may be more critical than reducing circulating sFlt-1 for improving endothelial function in preeclampsia. Consequently, further mechanistic studies are needed to clarify the complex interaction between antiangiogenic factors and NO signaling in the pathophysiology of preeclampsia.

Although VEGF levels and the sFlt-1/VEGF ratio did not differ significantly among all groups, a trend for the sFlt-1/VEGF ratio to rise in the RUPP group was observed. Even modest increases in this ratio may reflect a shift toward an anti-angiogenic profile, potentially contributing to the endothelial dysfunction. Such an imbalance has been consistently associated with increased vascular resistance, higher blood pressure levels, and greater disease severity in preeclampsia [35]. This pattern is in line with findings by Kaitu’u-Lino et al. (2018), who reported that ESO can reduce sFlt-1 secretion in vitro, although clinical data have shown limited efficacy *in vivo* [36]. Thus, our results support the emerging view that, despite promising cellular effects, ESO is insufficient to meaningfully modulate systemic angiogenic imbalance in ischemia-driven models such as RUPP.

In addition, genetic evidence supports the involvement of angiogenesis-related genes in preeclampsia. Single-nucleotide polymorphisms in *FLT1* and *VEGF* have been associated with increased susceptibility to the disorder, and genome-wide association studies have identified variants located near *FLT1* that are linked to preeclampsia [37]. These findings further support the central role of the VEGF–sFlt-1 axis in the pathophysiology of the disease and suggest that genetic variation within this pathway may contribute to its development [37].

sFlt-1 is an alternative splicing variant of VEGF-R1 pre-mRNA that generates a truncated soluble form of the receptor lacking the transmembrane domain, while retaining the six N-terminal immunoglobulin-like (Ig) domains (D1–D6) of the Flt-1 and the VEGF/PlGF-binding region. Since it is known that D2 alone can bind VEGF and that D2 and D3 are necessary and sufficient for VEGF binding, we selected the crystal structures of Flt-1 D2-VEGF (PDB ID 1FLT) [38], Flt-1 D2-D3-VEGF (extracted from PDB ID 5T89), as well as the Flt-1 D1-D6 (PDB ID 5T89) [39] for docking studies. Thus, to investigate whether the reduction in circulating sFlt-1 observed experimentally could be related to a direct interaction between ESO and the sFlt-1, we performed MD using GRAMM software [40] followed by 300 ns of unrestrained MD simulations using different sFlt-1 constructs.

Our docking and MD simulations analyses indicate that ESO does not form stable interactions with the VEGF-binding domains of sFlt-1, nor does it interfere with the sFlt-1/VEGF interface in any of the structural models evaluated. Although initial docking poses suggested weak and transient contacts, the ligand consistently dissociated from all predicted binding sites early during MD simulations, including the full-length extracellular D1–D6 construct. These findings indicate that the experimentally observed reduction in circulating sFlt-1 induced by ESO is unlikely to be mediated through perturbation of VEGF interaction or by affecting the stability of the sFlt-1 dimer interface, whose formation was shown to be induced by VEGF binding [39]. Instead, our computational data suggest that ESO likely acts through an alternative mechanism, potentially influencing sFlt-1 production, processing, or secretion rather than on VEGF binding. Therefore, the present computational results contradict the hypothesis of a direct interaction. Furthermore, giving support to the present results in RUPP+ESO rats and providing a mechanistic explanation for how ESO reduces sFlt-1 levels, previous studies have demonstrated that ESO upregulates heme-oxygenase 1 (HO-1)—a key placental protective enzyme—which may decrease sFlt-1 secretion [41,42]. Therefore, further mechanistic studies are warranted to clarify how ESO targets sFlt-1 *in vivo*.

Significant changes in placental weight were also detected among treated and untreated groups. Notably, placental efficiency—calculated as the fetal-to-placental weight ratio—was reduced in the RUPP, RUPP+ESO, and Preg+ESO groups. Reduced placental efficiency is generally interpreted as diminished functional performance of the placenta relative to its mass, reflecting a less favorable balance between placental growth and fetal demand. Lower placental efficiency in ESO-treated groups suggests that, despite preserved fetal weight, the placenta may have undergone adaptive or compensatory changes to sustain fetal development [43]. In this context, maintenance of fetal weight alongside reduced efficiency could indicate increased placental workload rather than improved functional capacity. Taken together, these findings underscore the complexity of placental adaptations in this model and highlight the need for detailed structural and molecular investigations to determine whether these changes represent compensatory remodeling, ongoing placental dysfunction, or potential drug-related effects on placental development.

Cautions regarding the interpretations of the present results should be considered. Although ESO decreased circulating sFlt-1 levels, the precise molecular pathways and mechanistic insights involved remain to be further clarified. Moreover, the use of a single experimental model may not completely represent the multifactorial pathophysiology of human preeclampsia. Further mechanistic and translational investigations are needed to explain the absence of functional improvement despite biochemical changes. Also, the precise contribution of each suppression of the nitrate–nitrite–NO pathway mechanism remains to be determined. Preeclampsia pathophysiology involves multiple converging mechanisms—including oxidative stress, inflammation, ADMA accumulation, and impaired NO signaling—that may persist independently of partial angiogenic correction. Additionally, our study does not provide direct mechanistic evidence explaining how ESO reduces sFlt-1.

## 4. Materials and Methods

### 4.1. Animals

The experimental protocol was approved by the Institutional Animal Care and Use Committee (protocol no. 6707090320) of the Institute of Biosciences of Botucatu, Sao Paulo State University. All procedures were conducted in accordance with the Animal Research: Reporting of In Vivo Experiments (ARRIVE) guidelines.

Female Wistar rats (12–16 weeks old, 200–250 g) were obtained from Anilab (Paulinia, SP, Brazil) and housed under controlled environmental conditions (12 h light/dark cycle, 22 ± 2 °C) with ad libitum access to standard chow (Nuvilab^®^, Quimtia^®^, Colombo, PR, Brazil) and potable water.

Animals were acclimated for at least one week prior to experimental procedures. For mating, two female rats were housed overnight with a fertile male in a 2:1 ratio (harem system). The following morning, a vaginal smear was performed and examined under light microscopy. The presence of spermatozoa and/or estrous-phase epithelial cells was considered as pregnancy day 1.

### 4.2. RUPP Model of Preeclampsia and Experimental Protocol

Pregnant rats in the RUPP and RUPP+ESO groups underwent the RUPP surgical model of preeclampsia on pregnancy day 14, as previously described [17]. Briefly, animals were anesthetized with isoflurane (2–3%), and a midline abdominal incision was performed under sterile conditions. To reduce blood flow to the uteroplacental circulation by approximately 40% [17], three silver clips were placed in the following vascular segments: one clip (internal diameter of 0.203 mm) on the lower abdominal aorta (above the iliac bifurcation), and two other clips (internal diameter of 0.100 mm) on the right and left branches of the ovarian arteries. The abdominal cavity was then sutured in layers, and animals received postoperative analgesia (Profenid^®^, Sanofi-Aventis^®^, São Paulo, SP, Brazil, 5 mg/kg/mL), following the standard protocols for animal use.

On the same day (pregnancy day 14), animals in the Preg and Preg+ESO groups underwent a Sham procedure in which similar abdominal incisions and suturing without clip placement were performed.

Pregnant rats were randomly assigned to four experimental groups (*n* = 8–10 per group):

Preg: Pregnant rats receiving saline.

Preg+ESO: Pregnant rats treated with sodium esomeprazole.

RUPP: RUPP model rats receiving saline.

RUPP+ESO: RUPP model rats treated with sodium esomeprazole.

Sodium esomeprazole (Ranbaxy Laboratories Ltd., New Delhi, India) was administered by oral gavage at a dose of 3.5 mg/kg/day once daily from gestational day 14 to 21. Control groups received an equivalent volume of saline as the vehicle. Drug solutions were freshly prepared daily and administered immediately

### 4.3. Maternal Blood Pressure Measurements

Maternal blood pressure was measured on gestational days 11 and 13 using non-invasive tail-cuff plethysmography (Insight, Ribeirao Preto, SP, Brazil; catalog #EFF-306). Each pregnant rat was pre-warmed in a heated chamber (Insight, Ribeirao Preto, SP, Brazil; #EFF-307) at 37 °C for 10 min to promote tail vasodilation. At least three cycles of cuff inflation/deflation by a trained operator were obtained per animal, and the mean value was calculated. These baseline measurements (before RUPP surgery) were within physiological limits and did not differ among all groups (data not shown).

On gestational day 21, rats were anesthetized with 2% isoflurane. A polyethylene catheter (PE-50, #2270835, Thermo Fisher Scientific, Waltham, MA, USA) filled with heparinized saline (Hemofol^®^, Cristália^®^, Itapira, SP, Brazil; 5000 IU/mL) was inserted into the left carotid artery and advanced toward the aortic arch for direct measurement of arterial pressure. The catheter was exteriorized and sealed with pin plugs. Following the surgical procedure, anesthesia was discontinued, and animals were allowed to recover for 30 min in a temperature-controlled environment to ensure hemodynamic stabilization. The catheter was connected to a pressure transducer coupled to a data acquisition system (MP150CE, Biopac Systems Inc., Goleta, CA, USA). Systolic and diastolic blood pressures, heart rate, and mean arterial pressure were recorded using AcqKnowledge software version 3.2.1 (Biopac Systems Inc., Goleta, CA, USA) continuously for at least 20 min after a stabilization period.

### 4.4. Euthanasia, Blood Collection, and Tissue Harvest

On pregnancy day 21, animals were anesthetized with isoflurane and euthanized by exsanguination via cardiac puncture for blood collection, followed by heart removal. Blood samples were collected into lyophilized heparin tubes (Vacutainer, Becton Dickinson, Oxford, UK) and centrifuged (10,000 rpm, 10 min, 4 °C) for plasma separation. Plasma samples were aliquoted and stored at –80 °C for biochemical analysis.

The thoracic aorta was carefully excised and placed in a cold Krebs solution for vascular reactivity studies.

A cesarean section was performed to collect fetuses and placentas following a midline laparotomy and uterine excision. Fetal and placental weights were individually recorded. The stomach was removed for gastric pH determination.

### 4.5. Fetal and Placental Parameters

The total number of fetuses per dam was recorded, and individual fetal and placental weights were measured. Placental efficiency was calculated as the ratio of fetal weight to placental weight, reflecting the functional capacity of the placenta to support fetal growth. All weight measurements were expressed in grams (g).

### 4.6. Gastric pH Analysis

Gastric pH was measured immediately after stomach excision. Gastric contents were gently collected by opening the lower esophagus and rinsing the gastric contents. The pH was determined using a calibrated microelectrode connected to a benchtop pH meter (OHAUS, model STATER 2200, Barueri, SP, Brazil), according to previously validated protocols, as described by Pinheiro [44].

### 4.7. Determination of sFlt-1, VEGF, and ADMA Biomarkers

Plasma concentrations of sFlt-1 (#MBS 2602003) and VEGF (#MBS355365, MyBioSource, San Diego, CA, USA) and ADMA (#EK247273, Elabscience Ltd., Wuhan, Hubei, China) were quantified using commercially available enzyme-linked immunosorbent assay (ELISA) kits, according to the manufacturers’ instructions.

Briefly, all reagents and samples were brought to room temperature prior to analysis. Assays were performed in 96-well microplates (Thermo Fisher Scientific, Waltham, MA, USA) pre-coated with capture antibodies specific for each analyte. A series of standards with known concentrations was prepared from the kit stock solution and added in duplicate to generate a standard curve covering the assay detection range. Plasma samples were appropriately diluted and added to the wells, allowing the target analytes to bind to the immobilized antibodies during incubation.

After washing to remove unbound substances, a biotinylated detection antibody was added, followed by streptavidin–horseradish peroxidase conjugate, according to the kit protocol. Wells were washed between each step to minimize nonspecific binding. Subsequently, a chromogenic substrate solution (TMB) was added and incubated in the dark for color development. The reaction was terminated with a stop solution, resulting in a yellow color proportional to the analyte concentration.

Absorbance was measured at 450 nm using a microplate spectrophotometer (Synergy 4, Biotek, Winooski, VT, USA). Concentrations were calculated from the standard curve using a four-parameter logistic (4-PL) regression model and corrected for sample dilution. All standards and samples were analyzed in duplicate, and internal quality controls were included to ensure assay reliability. sFlt-1, VEGF, and ADMA concentrations were expressed in pg/mL. The sFlt-1/VEGF ratio was also calculated to evaluate angiogenic balance.

### 4.8. Determination of NO Metabolites (NOx)

Plasma NOx levels were measured using the Griess reaction method, as previously described [45]. Briefly, plasma (190 µL) was deproteinized with zinc acetate (10 µL, 300 g/L) and centrifuged (10,000× *g* at 4 °C for 30 min). Plasma supernatant (50 µL) was transferred and incubated in a 96-well microplate with 100 μL of vanadium chloride III (5%, *m*/*v;* 208272, Sigma, St. Louis, MO, USA), 50 μL of 0.1% N-(1-naphthyl)-ethylenediamine dihydrochloride, NED, (*v*/*v;* N9125, Sigma, St. Louis, MO, USA), and 50 μL sulfanilamide (2%, *m*/*v;* S9251, Sigma, St. Louis, MO, USA). The reaction mixture was incubated at 37 °C for 3 h under gentle agitation and protected from light (thermomixer; Eppendorf, Darmstadt, Germany). Sodium nitrite standard solutions (1.56–100 µmol/L) prepared in deionized water and processed under identical conditions were used to generate the calibration curve. Absorbance at 535 nm was read on a microplate spectrophotometer (Synergy 4, Biotek, Winooski, VT, USA). NOx concentrations were expressed in µmol/L.

### 4.9. Vascular Reactivity

Vascular reactivity was assessed as described previously [46]. The thoracic aorta was dissected under a microscope (SZO-T, Optika, Ponteranica, Italy). Thoracic aorta rings were prepared as follows: two rings (3–4 mm in length) with intact endothelium and two rings in which the endothelium was mechanically removed.

Each ring was mounted with two wire hooks in a 10 mL organ bath containing Krebs–Henseleit solution (composition in mmol/L: NaCl 130; KCl 4.7; CaCl_2_ 1.6; KH_2_PO_4_ 1.2; MgSO_4_ 1.2; NaHCO_3_ 15; glucose 11.1). One hook was fixed, the other connected to an isometric force transducer (FORT10, Transbridge 4M, World Precision Instruments, WPI, Sarasota, FL, USA). Data were recorded and analyzed using AcqKnowledge software (v3.5.7, MP100, Biopac Systems Inc., Goleta, CA, USA).

Rings were equilibrated for 45 min (at 37 °C) under 1.5 g resting tension, and the solution was renewed every 15 min. A control contraction was induced by KCl (96 mM; P5405, Sigma, St. Louis, MO, USA), and rings were washed three times afterward. Resting tension remained stable throughout the stabilization period.

To examine endothelium-dependent relaxation, intact or denuded rings were pre-contracted (P6126, Sigma, St. Louis, MO, USA) with Phe (10^−6^ M), followed by cumulative additions of acetylcholine (A6625, Sigma, St. Louis, MO, USA) (ACh; 10^−9^ to 10^−4^ M).

Individual concentration–relaxation response curves were analyzed using nonlinear regression, and sigmoidal dose–response curves were fitted using the least squares method. The effective concentration that produced half the maximal response (EC_50_) was calculated and expressed as pEC_50_ (−log M). ACh-induced relaxations were expressed as % relaxation of Phe-induced contraction, as previously described [47,48].

### 4.10. Molecular Docking and Molecular Dynamics (MD) Simulations

The crystal structures of Flt-1 D2-VEGF (PDB ID 1FLT) [40], Flt-1 D2-D3-VEGF (extracted from PDB 5T89), and Flt-1 D1-D6 (PDB ID 5T89) were used for docking studies. MD was then performed between these structures and ESO (coordinates obtained at PubChem) using GRAMM software [40]. The D2 and D2–D3 structures were evaluated both in the presence and absence of VEGF, whereas the D1–D6 structure was analyzed only in the presence of VEGF. The best complexes were selected by Gibbs free energy expressed in Kcal/mol. Molecular dynamics (MD) simulations of the selected complexes were performed using GROMACS v.5.0.5 [49] using the CHARMM36m force field [50]. Initial input parameters were generated with the CHARMM-GUI web-server version 3.8 [51], and the protonation states of the residues were assigned to pH 7.4 using the PROPKA3 server version 3.5.1 [52]. Systems were constructed in a cubic box extending 10 Å from the outermost atoms, solvated with TIP3 water molecules, and equilibrated with 0.1 M of NaCl. Energy minimization was performed using the Steepest Descent algorithm until reaching energies below 100 kJ/mol/nm. Position restraints were then applied to the protein backbone and to the hydrogen atoms of both the protein and ligand. A 1 ns constant number, volume and temperature (NVT) ensemble was performed, generating the velocities randomly according to Maxwell-Boltzmann distribution at a temperature of 310 K using the V-rescale thermostat [52], followed by a 1 ns constant number, pressure and temperature (NPT) ensemble with Berendsen barostat [53] at 1 bar. Thereafter, an unrestrained 300 ns NPT step was conducted using the Nosé–Hoover thermostat [54,55] and Parrinello–Rahman barostat [56]. Short-range cutoffs for electrostatic and Van der Waals interactions were set to 12 Å with a force-switch function applied from 10 to 12 Å. Hydrogen bonds were constrained using the LINCS algorithm [57]. The analysis of the interaction between the protein and ESO after MD simulations was performed using tools available in VMD version 1.9.4a57 [58], and all structural figures were generated using PyMOL version 4.6.

### 4.11. Statistical Analysis

Statistical analysis was performed using GraphPad Prism^®^ (version 8.0.1, San Diego, CA, USA). Data normality was assessed using the Shapiro–Wilk test. We assessed hemodynamic, fetal, placental, and biochemical parameters by Two-way analysis of variance (ANOVA) followed by Sidak’s post hoc test. Two-way repeated-measures ANOVA followed by Sidak’s post hoc test was used to examine the differences at the acetylcholine concentrations (point-by-point) in vascular reactivity experiments. A *p*-value < 0.05 was considered statistically significant. Results are expressed as mean ± standard error of the mean (SEM).

## 5. Conclusions

The present study indicates that ESO may disturbs NO signaling and increases maternal blood pressure in normal pregnancy. ESO treatment reduces circulating sFlt-1 levels, but without changing the increases in maternal blood pressure and endothelial dysfunction induced by the RUPP model of preeclampsia in rats. These findings suggest that restoring NO signaling may be more relevant than reducing circulating sFlt-1 to improve outcomes in preeclampsia.

## Figures and Tables

**Table 1:** Maternal, fetal, placental, vascular, and biochemical parameters from the Preg, Preg+ESO, RUPP, and RUPP+ESO groups.

Parameter	Preg	Preg+ESO	RUPP	RUPP+ESO	*p* Interaction	*p* Treatment Effect	*p* Surgery Effect
Systolic blood pressure (mmHg)	102 ± 2	125 ± 3 *	126 ± 6 *	128 ± 3 *	0.0207	0.0088	0.0038
Diastolic blood pressure (mmHg)	92 ± 4	112 ± 3 *	111 ± 6 *	113 ± 4 *	0.0511	0.0184	0.0228
Mean arterial pressure (mmHg)	98 ± 4	120 ± 3 *	119 ± 7 *	123 ± 3 *	0.0783	<0.0001	0.0006
Heart rate (bpm)	341 ± 3	373 ± 2	349 ± 8	397 ± 7 *^#^	0.4629	0.0009	0.1484
Fetal weight (g)	4.00 ± 0.10	3.89 ± 0.06 ^#^	3.24 ± 0.07 *	3.58 ± 0.09 *	0.0096	0.2042	<0.0001
Number of pups	11 ± 1	11 ± 1	9 ± 1 *	10 ± 1	0.6723	0.9693	0.0007
Placental weight (g)	0.32 ± 0.004	0.46 ± 0.010 *^#^	0.29 ± 0.004 *	0.41 ± 0.008 *^#^	0.7205	<0.0001	<0.0001
Placental efficiency	13 ± 0.4	12 ± 0.3	8.8 ± 0.1	8.3 ± 0.3	0.1589	<0.0001	0.0049
Stomach pH (−log[H^+^])	4 ± 0.1	5 ± 0.3 *^#^	4 ± 0.1	5 ± 0.4 *^#^	0.9812	<0.0001	0.6421
sFlt-1 (pg/mL)	1172 ± 62	1039 ± 53 ^#^	1847 ± 100 *	1216 ± 72 ^#^	0.0028	<0.0001	<0.0001
VEGF (pg/mL)	40 ± 7	44 ± 6	35 ± 2	28 ± 2	0.3462	0.7697	0.0575
sFlt-1/VEGF ratio	36 ± 7.2	35 ± 8.2	53 ± 4.5	41 ± 4.8	0.3783	0.3531	0.0741
ADMA (pg/mL)	49 ± 5	47 ± 6	143 ± 18 *	118 ± 16 *	0.3640	0.2868	<0.0001
Plasma NO metabolites (mmol/L)	116 ± 8	89 ± 2 *	83 ± 2 *	79 ± 4 *	0.0416	0.0093	0.0004
Acetylcholine with intact endothelium *E_max_* (%)	80 ± 6	64 ± 3 *	53 ± 6 *	61 ± 10 *	0.9998	0.0696	0.7337
Acetylcholine with intact endothelium pEC_50_ (−log M)	5.0 ± 0.2	6.0 ± 0.5	5.0 ± 0.2	6.0 ± 0.2	0.6059	0.0589	0.5513

Data are expressed as mean ± SEM. Preg, Preg+ESO, RUPP, and RUPP+ESO groups included 8–10 animals per group. * *p* < 0.05 vs. Preg and ^#^ *p* < 0.05 vs. RUPP group. Statistical analysis was performed using two-way ANOVA considering surgery (Preg vs. RUPP) and treatment (vehicle vs. esomeprazole) as factors. A value of *p* < 0.05 was considered statistically significant. ESO—esomeprazole; RUPP—reduced uterine perfusion pressure; NO—nitric oxide; sFlt-1—soluble fms-like tyrosine kinase-1; VEGF—vascular endothelial growth factor; ADMA—asymmetric dimethylarginine.

## Data Availability

The original contributions presented in this study are included in the article/Supplementary Materials. Further inquiries can be directed to the corresponding author.

## Supplementary Materials

The following supporting information can be downloaded at https://www.mdpi.com/article/10.3390/ijms27073105/s1.

## References

[1] Ma’ayeh M., Costantine M.M. (2020). Prevention of Preeclampsia. Semin. Fetal Neonatal Med..

[2] Albrecht E.D., Pepe G.J. (2020). Regulation of Uterine Spiral Artery Remodeling: A Review. Reprod. Sci..

[3] de Alwis N., Binder N.K., Beard S., Kaitu’U-Lino T.J., Tong S., Brownfoot F., Hannan N.J. (2020). Novel approaches to combat preeclampsia: From new drugs to innovative delivery. Placenta.

[4] Sutton E.F., Gemmel M., Powers R.W. (2020). Nitric Oxide Signaling in Pregnancy and Preeclampsia. Nitric Oxide.

[5] Maynard S.E., Min J.-Y., Merchan J., Lim K.-H., Li J., Mondal S., Libermann T.A., Morgan J.P., Sellke F.W., Stillman I.E. (2003). Excess Placental Soluble Fms-like Tyrosine Kinase 1 (sFlt1) May Contribute to Endothelial Dysfunction, Hypertension, and Proteinuria in Preeclampsia. J. Clin. Investig..

[6] Levine R.J., Maynard S.E., Qian C., Lim K.-H., England L.J., Yu K.F., Schisterman E.F., Thadhani R., Sachs B.P., Epstein F.H. (2004). Circulating Angiogenic Factors and the Risk of Preeclampsia. N. Engl. J. Med..

[7] Cindrova-Davies T., Sanders D.A., Burton G.J., Charnock-Jones D.S. (2011). Soluble FLT1 sensitizes endothelial cells to inflammatory cytokines by antagonizing VEGF receptor-mediated signalling. Cardiovasc. Res..

[8] Shabana H., Cesta C.E., Yan J., Brusselaers N., Rodriguez-Wallberg K.A. (2025). Proton pump inhibitors and the risk of infection during pregnancy and postpartum: A population-based register cohort study in Sweden. Lancet Obstet. Gynecol. Women’s Health.

[9] de Alwis N., Binder N.K., Mangwiro Y.T.M., Beard S., Pritchard N., Kadife E., Fato B.R., Keenan E., Brownfoot F.C., Kaitu’u-Lino T.J. (2022). Actions of Esomeprazole on the Maternal Vasculature in Lean and Obese Pregnant Mice with Impaired Nitric Oxide Synthesis: A Model of Preeclampsia. Int. J. Mol. Sci..

[10] Pasternak B., Hviid A. (2010). Use of Proton-Pump Inhibitors in Early Pregnancy and the Risk of Birth Defects. N. Engl. J. Med..

[11] Vachhani R., Olds G., Velanovich V. (2009). Esomeprazole: A Proton Pump Inhibitor. Expert Rev. Gastroenterol. Hepatol..

[12] Matok I., Levy A., Wiznitzer A., Uziel E., Koren G., Gorodischer R. (2012). The Safety of Fetal Exposure to Proton-Pump Inhibitors During Pregnancy. Dig. Dis. Sci..

[13] Lundberg J.O., Weitzberg E., Gladwin M.T. (2008). The Nitrate–Nitrite–Nitric Oxide Pathway in Physiology and Therapeutics. Nat. Rev. Drug Discov..

[14] Zhang K., Li J., Wang G., Cao Y., Yang F., You T., Liu C., Li M., Hu S., Ren L. (2023). Impact of gastric acid suppression on nitrate–nitrite–NO pathway and cardiovascular homeostasis. Cardiovasc. Res..

[15] Sandrim V.C., Caldeira-Dias M., Montenegro M.F. (2019). Esomeprazole to treat women with preeclampsia: Possible implications in the nitric oxide homeostasis. Am. J. Obstet. Gynecol..

[16] Mills K., McDougall A., Tan A., Makama M., Nguyen P., Armari E., Bradfield Z., Hastie R., Ammerdorffer A., Gülmezoglu A. (2024). The effects of proton pump inhibitors during pregnancy on treatment of preeclampsia and related outcomes: A systematic review and meta-analysis. Am. J. Obstet. Gynecol. MFM.

[17] Li J., LaMarca B., Reckelhoff J.F. (2012). A Model of Preeclampsia in Rats: The Reduced Uterine Perfusion Pressure (RUPP) Model. Am. J. Physiol.-Heart Circ. Physiol..

[18] Soliman A.I., Wactawski-Wende J., Millen A.E., Gray S.L., Eaton C.B., Hovey K.M., Donneyong M., Saquib N., Mouton C.P., Laddu D. (2025). Proton Pump Inhibitor Use and Incident Hypertension in Menopausal Women. J. Am. Heart Assoc..

[19] Kapil V., Milsom A.B., Okorie M., Maleki-Toyserkani S., Akram F., Rehman F., Arghandawi S., Pearl V., Benjamin N., Loukogeorgakis S. (2010). Inorganic Nitrate Supplementation Lowers Blood Pressure in Humans. Hypertension.

[20] Webb A.J., Patel N., Loukogeorgakis S., Okorie M., Aboud Z., Misra S., Rashid R., Miall P., Deanfield J., Benjamin N. (2008). Acute Blood Pressure Lowering, Vasoprotective, and Antiplatelet Properties of Dietary Nitrate via Bioconversion to Nitrite. Hypertension.

[21] Ghebremariam Y.T., LePendu P., Lee J.C., Erlanson D.A., Slaviero A., Shah N.H., Leiper J., Cooke J.P. (2013). Unexpected Effect of Proton Pump Inhibitors. Circulation.

[22] Montenegro M.F., Sundqvist M.L., Larsen F.J., Zhuge Z., Carlström M., Weitzberg E., Lundberg J.O. (2017). Blood Pressure–Lowering Effect of Orally Ingested Nitrite Is Abolished by a Proton Pump Inhibitor. Hypertension.

[23] Röhss K., Hasselgren G., Hedenström H. (2002). Effect of Esomeprazole 40 Mg vs Omeprazole 40 Mg on 24-Hour Intragastric pH in Patients with Symptoms of Gastroesophageal Reflux Disease. Dig. Dis. Sci..

[24] Katz P., Kahrilas P.J., Johnson D.A., Lind T., Röhss K., Traxler B., Hugo V., Dent J. (2015). Daytime Intragastric Acid Control: Post Hoc Analyses of Esomeprazole 20 Mg and over-the-Counter Proton-Pump Inhibitors. Therap. Adv. Gastroenterol..

[25] Justina V.D., Dos Passos Júnior R.R., Lima V.V., Giachini F.R. (2023). Evidence of nitric oxide impairment during hypertensive pregnancies. Adv. Exp. Med. Biol..

[26] Zanzinger J. (1999). Role of Nitric Oxide in the Neural Control of Cardiovascular Function. Cardiovasc. Res..

[27] Arafah A.M., Ahmad A., Jan B.L., Maghawi K.M., Alharbi M.A., Alkharfy K.M. (2018). Pantoprazole Reduces Vascular Relaxation in-Vitro and Ex-Vivo and Interferes with Blood Coagulation in an Animal Model. Biomed. Pharmacother..

[28] Cziráki A., Lenkey Z., Sulyok E., Szokodi I., Koller A. (2020). L-Arginine-Nitric Oxide-Asymmetric Dimethylarginine Pathway and the Coronary Circulation: Translation of Basic Science Results to Clinical Practice. Front. Pharmacol..

[29] Willeit P., Freitag D.F., Laukkanen J.A., Chowdhury S., Gobin R., Mayr M., Di Angelantonio E., Chowdhury R. (2015). Asymmetric dimethylarginine and cardiovascular risk: Systematic review and meta-analysis of 22 prospective studies. J. Am. Heart Assoc..

[30] Németh B., Murányi E., Hegyi P., Mátrai P., Szakács Z., Varjú P., Hamvas S., Tinusz B., Budán F., Czimmer J. (2018). Asymmetric Dimethylarginine Levels in Preeclampsia—Systematic Review and Meta-Analysis. Placenta.

[31] Tommasi S., Elliot D.J., Hulin J.A., Lewis B.C., McEvoy M., Mangoni A.A. (2017). Human Dimethylarginine Dimethylaminohydrolase 1 Inhibition by Proton Pump Inhibitors and the Cardiovascular Risk Marker Asymmetric Dimethylarginine: In Vitro and in Vivo Significance. Sci. Rep..

[32] Burwick R.M., Fichorova R.N., Dawood H.Y., Yamamoto H.S., Feinberg B.B. (2013). Urinary Excretion of C5b-9 in Severe Preeclampsia. Hypertension.

[33] Saleh L., Samantar R., Garrelds I., van den Meiracker A., Visser W., Danser A. (2017). Low soluble fms-like tyrosine kinase-1, endoglin, and endothelin-1 levels in women with confirmed or suspected preeclampsia using proton pump inhibitors. Hypertension.

[34] Dymara-Konopka W., Laskowska M. (2019). The Role of Nitric Oxide, ADMA, and Homocysteine in The Etiopathogenesis of Preeclampsia-Review. Int. J. Mol. Sci..

[35] Ives C.W., Sinkey R., Rajapreyar I., Tita A.T.N., Oparil S. (2020). Preeclampsia—Pathophysiology and clinical presentations: JACC State-of-the-Art Review. J. Am. Coll. Cardiol..

[36] Kaitu’u-Lino T.J., Brownfoot F.C., Beard S., Cannon P., Hastie R., Nguyen T.V., Binder N.K., Tong S., Hannan N.J. (2018). Combining Metformin and Esomeprazole Is Additive in Reducing sFlt-1 Secretion and Decreasing Endothelial Dysfunction—Implications for Treating Preeclampsia. PLoS ONE.

[37] McGinnis R., Steinthorsdottir V., Williams N.O., Thorleifsson G., Shooter S., Hjartardottir S., Bumpstead S., Stefansdottir L., Hildyard L., Sigurdsson J.K. (2017). Variants in the fetal genome near FLT1 are associated with risk of preeclampsia. Nat Genet..

[38] Wiesmann C., Fuh G., Christinger H.W., Eigenbrot C., Wells J.A., Vos A.M. (1997). de Crystal Structure at 1.7 Å Resolution of VEGF in Complex with Domain 2 of the Flt-1 Receptor. Cell.

[39] Markovic-Mueller S., Stuttfeld E., Asthana M., Weinert T., Bliven S., Goldie K.N., Kisko K., Capitani G., Ballmer-Hofer K. (2017). Structure of the Full-Length VEGFR-1 Extracellular Domain in Complex with VEGF-A. Structure.

[40] Singh A., Copeland M.M., Kundrotas P.J., Vakser I.A., Gore M., Jagtap U.B. (2024). GRAMM Web Server for Protein Docking. Computational Drug Discovery and Design.

[41] Cudmore M., Ahmad S., Al-Ani B., Fujisawa T., Coxall H., Chudasama K., Devey L.R., Wigmore S.J., Abbas A., Hewett P.W. (2007). Negative regulation of soluble Flt-1 and soluble endoglin release by heme oxygenase-1. Circulation.

[42] Ebrahimpour A., Wang M., Li L., Jegga A.G., Bonnen M.D., Eissa N.T., Raghu G., Jyothula S., Kheradmand F., Hanania N.A. (2021). Esomeprazole attenuates inflammatory and fibrotic response in lung cells through the MAPK/Nrf2/HO1 pathway. J. Inflamm..

[43] Hayward C.E., Lean S.C., Sibley C.P., Jones R.L., Wareing M., Greenwood S.L., Dilworth M.R. (2016). Placental adaptation: What can we learn from birthweight:placental weight ratio?. Front. Physiol..

[44] Pinheiro L.C., Oliveira-Paula G.H., Portella R.L., Guimarães D.A., de Angelis C.D., Tanus-Santos J.E. (2016). Omeprazole Impairs Vascular Redox Biology and Causes Xanthine Oxidoreductase-Mediated Endothelial Dysfunction. Redox Biol..

[45] Tatsch E., Bochi G.V., Pereira R.d.S., Kober H., Agertt V.A., Anraku de Campos M.M., Gomes P., Duarte M.M.M.F., Moresco R.N. (2011). A Simple and Inexpensive Automated Technique for Measurement of Serum Nitrite/Nitrate. Clin. Biochem..

[46] Raffetto J.D., Calanni F., Mattana P., Khalil R.A. (2019). Sulodexide Promotes Arterial Relaxation via Endothelium-Dependent Nitric Oxide-Mediated Pathway. Biochem. Pharmacol..

[47] Zhu M., Ren Z., Possomato-Vieira J.S., Khalil R.A. (2016). Restoring Placental Growth Factor-Soluble Fms-like Tyrosine Kinase-1 Balance Reverses Vascular Hyper-Reactivity and Hypertension in Pregnancy. Am. J. Physiol.-Regul. Integr. Comp. Physiol..

[48] Da Silva M.L.S., Gomes S.E.B., Martins L.Z., Rodrigues S.D., Toghi C.d.J., Dias-Junior C.A. (2024). Impaired Endothelium-Dependent Vasodilation and Increased Levels of Soluble Fms-like Tyrosine Kinase-1 Induced by Reduced Uterine Perfusion Pressure in Pregnant Rats: Evidence of Protective Effects with Sodium Nitrite Treatment in Preeclampsia. Int. J. Mol. Sci..

[49] Abraham M.J., Murtola T., Schulz R., Páll S., Smith J.C., Hess B., Lindahl E. (2015). GROMACS: High Performance Molecular Simulations through Multi-Level Parallelism from Laptops to Supercomputers. SoftwareX.

[50] Huang J., MacKerell A.D. (2013). CHARMM36 All-Atom Additive Protein Force Field: Validation Based on Comparison to NMR Data. J. Comput. Chem..

[51] Olsson M.H.M., Søndergaard C.R., Rostkowski M., Jensen J.H. (2011). PROPKA3: Consistent Treatment of Internal and Surface Residues in Empirical pKa Predictions. J. Chem. Theory Comput..

[52] Bussi G., Donadio D., Parrinello M. (2007). Canonical Sampling Through Velocity Rescaling. J. Chem. Phys..

[53] Berendsen H.J.C., Postma J.P.M., van Gunsteren W.F., DiNola A., Haak J.R. (1984). Molecular Dynamics with Coupling to an External Bath. J. Chem. Phys..

[54] Nosé S. (1984). A Molecular Dynamics Method for Simulations in the Canonical Ensemble. Mol. Phys..

[55] Hoover W.G. (1985). Canonical Dynamics: Equilibrium Phase-Space Distributions. Phys. Rev. A.

[56] Parrinello M., Rahman A. (1981). Polymorphic Transitions in Single Crystals: A New Molecular Dynamics Method. J. Appl. Phys..

[57] Hess B., Bekker H., Berendsen H.J.C., Fraaije J.G.E.M. (1997). LINCS: A Linear Constraint Solver for Molecular Simulations. J. Comput. Chem..

[58] Humphrey W., Dalke A., Schulten K. (1996). VMD: Visual Molecular Dynamics. J. Mol. Graph..

