# Supplementary material for: Esomeprazole Decreases Soluble Fms-like Tyrosine Kinase-1 in Preeclamptic Pregnancy in Rats
Source: Int J Mol Sci. 2026 Mar 29;27(7):3105. doi: 10.3390/ijms27073105 (PMC13072813; doi:10.3390/ijms27073105)
Supplement: Supplementary file 1 [file ijms-27-03105-s001.zip › ijms-4117476-supplementary.pdf]

International Journal of
Molecular Sciences

MDPI

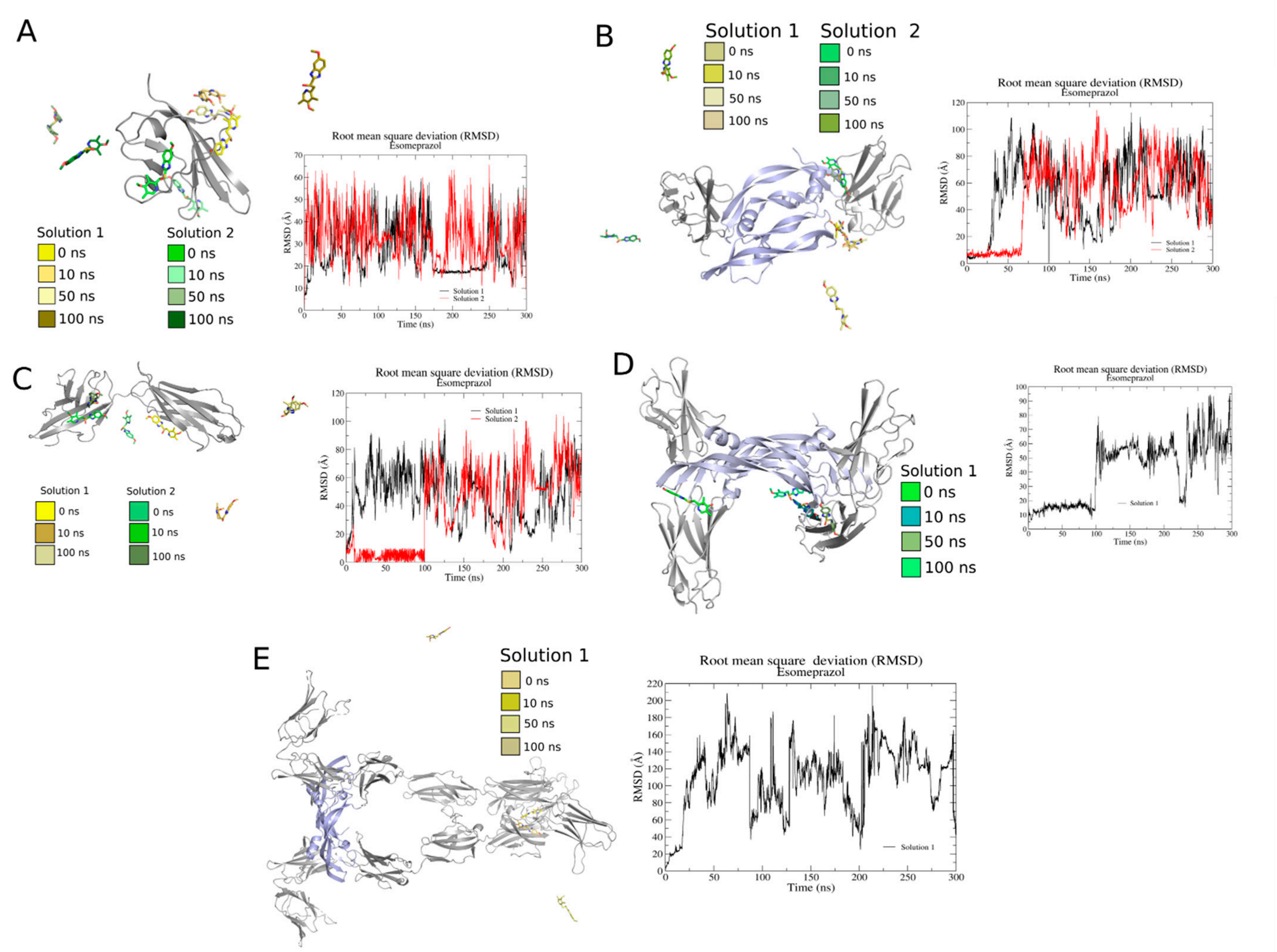

Supplementary Figure 1. Analysis of sFlt-1 interaction by molecular docking and molecular dynamics (MD) simulations. Docking poses of esomeprazole in the different sFlt-1 constructs and their stability during 300 ns of unrestrained MD simulations. (A) D2; (B) D2-VEGF; (C) D2-D3; (D) D2-D3-VEGF; (E) D1-D6. Each panel shows snapshots of esomeprazole positions along the MD trajectory, together with the root mean square deviation (RMSD) of the esomeprazole atoms throughout the simulation, which indicates how much the ligand moves relative to its initial docking pose. D2, D3 and D1-D6 from sFlt-1 are shown in gray cartoons and VEGF is shown in light purple cartoons.
